# Association of hydroxychloroquine and cardiac arrhythmia in patients with systemic lupus erythematosus: A population-based case control study

**DOI:** 10.1371/journal.pone.0251918

**Published:** 2021-05-20

**Authors:** Chien-Hsien Lo, Yu-Hsun Wang, Chin-Feng Tsai, Kuei-Chuan Chan, Li-Ching Li, Tse-Hsien Lo, James Cheng-Chung Wei, Chun-Hung Su

**Affiliations:** 1 Institute of Medicine, School of Medicine, Chung Shan Medical University, Taichung, Taiwan; 2 Division of Cardiology, Department of Internal Medicine, Chung-Shan Medical University Hospital, Taichung, Taiwan; 3 Department of Medical Research, Chung Shan Medical University Hospital, Taichung, Taiwan; 4 Department of Internal Medicine, Chung-Shan Medical University Hospital, Taichung, Taiwan; 5 Department of Internal Medicine, Da Chien General Hospital, Miaoli, Taiwan; 6 Department of Allergy, Immunology & Rheumatology, Chung Shan Medical University Hospital, Taichung, Taiwan; 7 Institute of Medicine, College of Medicine, Chung Shan Medical University, Taichung, Taiwan; 8 Graduate Institute of Integrated Medicine, China Medical University, Taichung, Taiwan; 9 Department of Medical Research, Taichung Veterans General Hospital, Taichung, Taiwan; Kaplan Medical Center, ISRAEL

## Abstract

**Objectives:**

Hydroxychloroquine is widely used to treat certain viral and rheumatic diseases including systemic lupus erythematosus. Cardiac arrhythmia is an important safety issue with hydroxychloroquine. The aim of this study was to investigate whether hydroxychloroquine increases new-onset arrhythmia among patients with systemic lupus erythematosus.

**Methods:**

This was a nested case-control study using data from the Longitudinal Health Insurance Database of Taiwan. A conditional logistic regression model was used to analyse differences in the risk of arrhythmia between systemic lupus erythematosus patients with and without hydroxychloroquine treatment after controlling for related variables.

**Results:**

A total of 2499 patients with newly diagnosed systemic lupus erythematosus were identified (81% females), of whom 251 were enrolled in the new-onset arrhythmia group (mean age 50.4 years) and 251 in the non-arrhythmia group (mean age 49.1 years). There was no significantly increased risk of cardiac arrhythmia (adjusted odds ratio = 1.49, 95% confidence interval: 0.98–2.25) or ventricular arrhythmia (adjusted odds ratio = 1.02, 95% confidence interval: 0.19–5.41) between the patients with and without hydroxychloroquine treatment. In addition, there were no significant differences in the risk of arrhythmia between those receiving hydroxychloroquine treatment for <180 days or ≥180 days, with a drug adherence rate of <50% or ≥50%, and receiving a daily dose of <400 mg or ≥400 mg.

**Conclusion:**

In patients with systemic lupus erythematosus, hydroxychloroquine treatment did not significantly increase the risk of cardiac arrhythmia or life-threatening ventricular arrhythmia regardless of the different hydroxychloroquine treatment duration, drug adherence rate, or daily dose.

## Introduction

The antimalarial drug hydroxychloroquine (HCQ) has been used as standard treatment to reduce disease activity and improve survival for certain inflammatory diseases such as systemic lupus erythematosus (SLE) and rheumatoid arthritis (RA) for more than half a century [1–3]. HCQ can modulate and protect against systemic inflammation and prothrombotic signalling pathways by inhibiting endosomal NADPH oxidase [4]. HCQ has also been demonstrated to reduce glycosylation of severe acute respiratory syndrome coronavirus (SARS-CoV) receptors that block the entry site via ACE2 preceptors [5, 6]. Coronavirus disease 2019 (Covid-19) is caused by SARS-CoV-2, which is transmitted rapidly and is widespread, and threatens the global population [7]. During the early outbreak, HCQ was used as a therapeutic option for Covid-19 treatment whether in combination with azithromycin or not [8, 9].

The risk of cardiac arrhythmias in patients receiving HCQ is an important safety issue. HCQ slows the rate of action potential firing in sinoatrial nodes through channels that inhibit the “funny” current (I_f_) [10]. It has also been shown to cause inhomogeneous lengthening of the action potential and prolongation of corrected QT interval (QTc) by inhibiting the rapid component of the delayed rectifier potassium current (iKr), which potentially results in life-threatening ventricular arrhythmias such as torsades de pointes (TdP) [11–13]. Previous reports have shown that the risk of cardiac arrhythmia may be increased in patients using HCQ [14, 15], however the evidence is limited and the results have been inconsistent. Moreover, in everyday practice, very few arrhythmic events have been reported in patients taking HCQ. Therefore, the aim of this study was to clarify whether the risk of arrhythmia is increased in patients receiving HCQ therapy for SLE using a large population-based dataset from the National Health Insurance Research Database (NHIRD) in Taiwan.

## Materials and methods

### Data source

This retrospective case control study was conducted using data from the Taiwan National Health Insurance (NHI) program, which covers approximately 99% of the population in Taiwan. The NHIRD contains information on outpatient visits, inpatient care, medical procedures, and medications. We used the Longitudinal Health Insurance Database (LHID) which contains one million people randomly sampled from the NHIRD with International Classification of Diseases, 9th Revision, Clinical Modification (ICD-9-CM) diagnosis codes [16]. All data were completely anonymized, deidentified and aggregated before we accessed them. The Research Ethics Committee of Chung Shan Medical University and Hospital approved this study (CS-17114) and patient consent for publication is not required.

### Patient selection

This study was conducted using claims data from 1999 to 2013 from the LHID. We enrolled all people who were newly diagnosed with SLE (ICD-9-CM = 710.0) [17, 18] with at least two outpatient clinic visits or one admission from 2000 to 2012. Patients with a diagnosis of arrhythmia (ICD-9-CM = 426, 427) before the diagnosis of SLE were excluded. The first date of newly diagnosed arrhythmia after a diagnosis of SLE was set as the index date. The comparison group included patients who had not been diagnosed with arrhythmia after a diagnosis of SLE. The study period was from the first date when SLE was diagnosed to the index date.

The main cardiac arrhythmia in this study was defined as a new diagnosis of all arrhythmias (ICD-9-CM = 426–427) [19], including conduction disorders such as atrioventricular block and bundle branch block (ICD-9-CM = 426), supraventricular tachyarrhythmias, ventricular tachyarrhythmias and sinus node dysfunction (ICD-9-CM = 427). Ventricular tachyarrhythmias alone (ICD-9-CM = 427.1, 427.4–427.5) were also analysed as sub-group data.

### Covariates and matching

To minimise the effect of confounding factors, we performed simple matching to obtain a 1:1 ratio by age, sex and year of SLE diagnosis. The analysed covariates were underlying comorbidities including hypertension (ICD-9-CM = 401–405), hyperlipidemia (ICD-9-CM = 272.0–272.4), chronic liver disease (ICD-9-CM = 571), chronic kidney disease (ICD-9-CM = 585), diabetes mellitus (ICD-9-CM = 250), chronic obstructive pulmonary disease (ICD-9-CM = 491, 492, 496), ischaemic heart disease (ICD-9-CM = 410–414), heart failure (ICD-9-CM = 428), stroke (ICD-9-CM = 430–438), and alcoholism (ICD-9-CM = 305, 571.0–571.2).

### Statistical analysis

To compare the characteristics of the patients with and without arrhythmias and HCQ therapy, the chi-square test was used for categorical variables and independent t test for continuous variables. We used a conditional logistic regression model to estimate crude odds ratios (ORs), adjusted ORs, and 95% confidence intervals (CIs) between the two groups. The per-day HCQ dosage was also calculated to analyse the risk of arrhythmia. All statistical analyses were conducted using SPSS version 18.0 (SPSS Inc., Chicago, IL, USA). The null hypothesis of no significant difference between two groups was rejected if P < 0.05.

## Results

**Fig 1:**
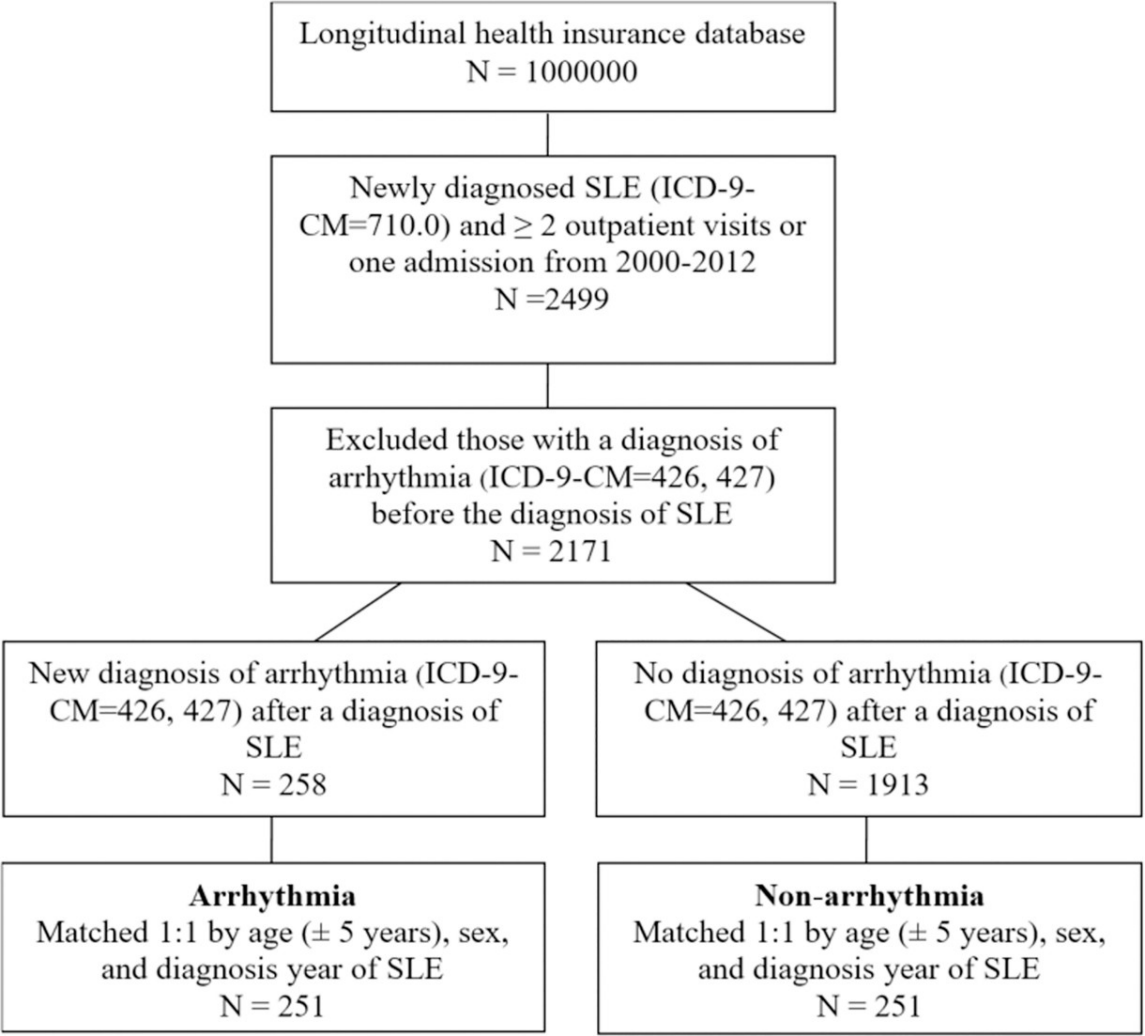
Flow chart of patient selection.

The flow chart of patient selection in this study is illustrated in Fig 1. Among the one million patients, 2499 with newly diagnosed SLE were selected to participate in the study. We excluded 328 patients who had arrhythmia before their SLE diagnosis, and the remaining patients with or without newly diagnosed arrhythmia were classified into two groups for further comparisons. After matching at a 1:1 ratio, a total of 251 patients were enrolled in each group.

The baseline patient demographic and clinical characteristics are summarised in Table 1. The mean (SD) age of the patients was 50.4 (17.9) years in the arrhythmia group and 49.1 (17.4) years in the non-arrhythmia group. Overall, 81% of the study population was female. Most underlying comorbidities were balanced after matching except for ischaemic heart disease and heart failure.

**Table 1:** Demographic characteristics of the SLE patients with and without arrhythmia.

	With arrhythmia (N = 251)		Without arrhythmia (N 251)		
	n	%	n	%	p-value
Age (years)					0.412
<50	118	47.0	131	52.2	
≥50	133	53.0	120	47.8	
Mean ± SD	50.4 ± 17.9		49.1 ± 17.4		0.412
Sex					1
Female	205	81.7	205	81.7	
Male	46	18.3	46	18.3	
Hypertension	87	34.7	69	27.5	0.083
Hyperlipidemia	51	20.3	40	15.9	0.203
Chronic liver disease	51	20.3	49	19.5	0.823
Chronic kidney disease	19	7.6	9	3.6	0.052
Diabetes mellitus	40	15.9	27	10.8	0.088
COPD	31	12.4	18	7.2	0.051
Ischaemic heart disease	54	21.5	19	7.6	<0.001
Heart failure	14	5.6	1	0.4	0.001
Stroke	28	11.2	18	7.2	0.122
Alcoholism	5	2.0	2	0.8	0.450†
Hydroxychloroquine	104	41.4	87	34.7	0.118
Diagnosis year of SLE					1
2000	60	23.9	60	23.9	
2001	34	13.5	34	13.5	
2002	31	12.4	31	12.4	
2003	25	10.0	25	10.0	
2004	25	10.0	25	10.0	
2005	15	6.0	15	6.0	
2006	18	7.2	18	7.2	
2007	14	5.6	14	5.6	
2008	13	5.2	13	5.2	
2009	6	2.4	6	2.4	
2010	5	2.0	5	2.0	
2011	2	0.8	2	0.8	
2012	3	1.2	3	1.2	
Study period (years)	4.6 ± 3.5		4.6 ± 3.5		0.995

COPD: chronic obstructive pulmonary disease. SLE: systemic lupus erythematosus

†Fisher’s exact test.

Table 2 shows the results of conditional logistic regression analysis to determine the ORs for arrhythmia. The patients who received HCQ therapy seemed to have a higher risk of arrhythmia, however the difference did not reach significance (adjusted OR 1.49 (95% CI 0.98–2.25, p = 0.060). The patients with underlying ischaemic heart disease had a significantly higher risk of arrhythmia (adjusted OR 4.82, 95% CI 2.16–10.74, p<0.001). The patients with underlying hypertension, chronic obstructive pulmonary disease and heart failure seemed to have higher risks of arrhythmia, however the differences did not reach significance after adjusting the ORs. There were no significant associations between other comorbidities and the risk of arrhythmia.

**Table 2:** Multivariable logistic regression analysis of factors associated with arrhythmia in the SLE patients.

	Crude OR	95% CI	p-value	Adjusted OR^†^	95% CI	p-value
Hydroxychloroquine	1.33	0.93–1.92	0.120	1.49	0.98–2.25	0.060
Hypertension	1.58	1.01–2.48	0.046	1.07	0.63–1.83	0.797
Hyperlipidemia	1.48	0.87–2.51	0.148	1.22	0.62–2.38	0.566
Chronic liver disease	1.07	0.65–1.76	0.800	0.87	0.48–1.58	0.648
Chronic kidney disease	2.25	0.98–5.17	0.056	2.42	0.86–6.85	0.096
Diabetes mellitus	1.65	0.95–2.88	0.077	1.54	0.78–3.02	0.210
COPD	2.00	1.03–3.89	0.041	1.72	0.80–3.70	0.164
Ischaemic heart disease	5.37	2.53–11.43	<0.001	4.82	2.16–10.74	<0.001
Heart failure	14.00	1.84–106.46	0.011	6.62	0.82–53.37	0.076
Stroke	1.71	0.89–3.31	0.109	1.44	0.64–3.24	0.382
Alcoholism	2.50	0.49–12.89	0.273	4.13	0.60–28.36	0.149

COPD: chronic obstructive pulmonary disease. SLE: systemic lupus erythematosus

†Adjusted for hydroxychloroquine, hypertension, hyperlipidemia, chronic liver disease, chronic kidney disease, diabetes mellitus, COPD, ischemic heart disease, stroke, alcoholism, and heart failure.

**Fig 2:**
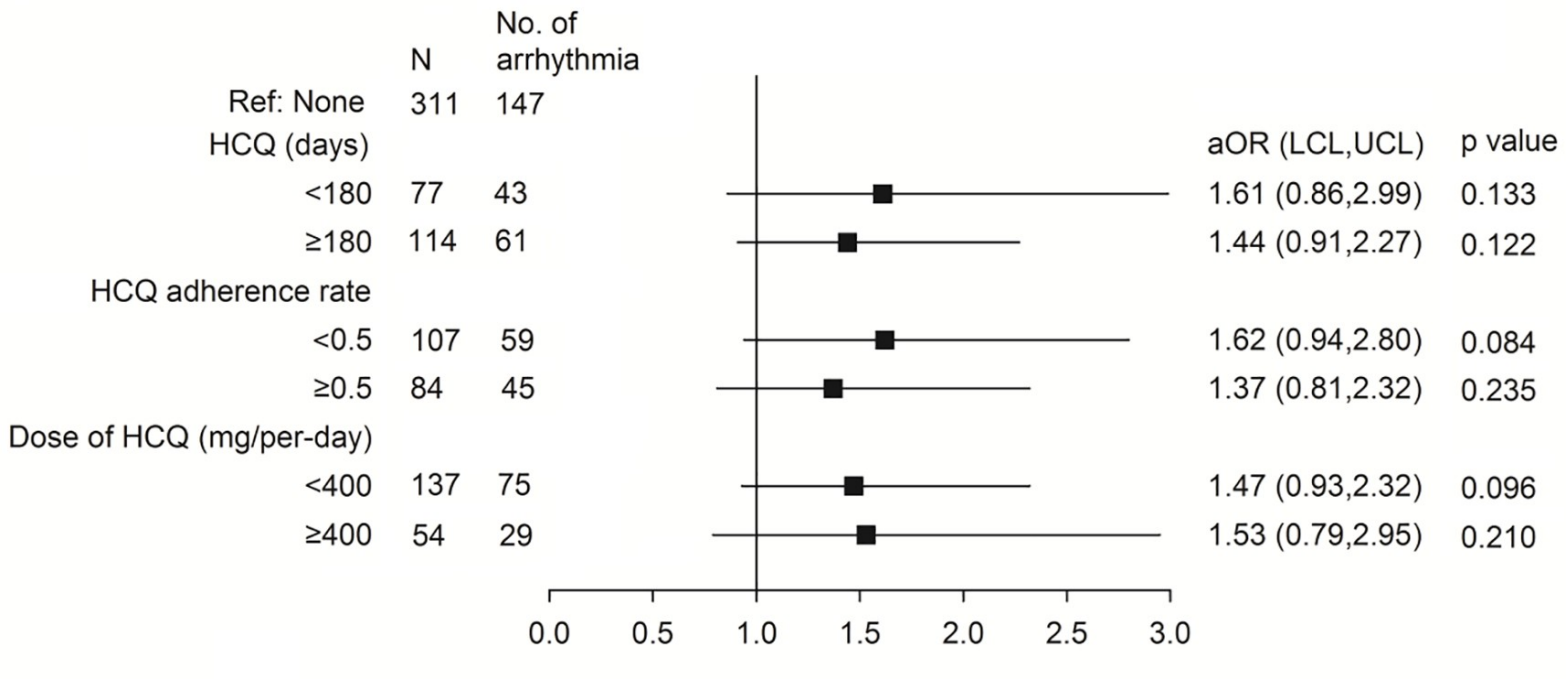
Dose response relationship of hydroxychloroquine for risk of arrhythmia.

**Fig 3:**
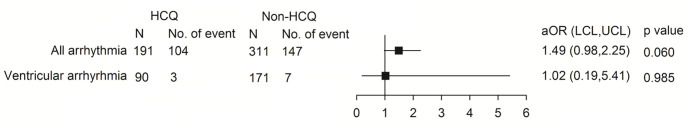
Sensitivity analysis of hydroxychloroquine for risk of ventricular arrhythmia and all kinds of arrhythmia.

The ORs for the relationships between arrhythmia and HCQ adherence and cumulative dose are shown in Fig 2. Compared to the non-HCQ group, the risk of arrhythmia did not increase with HCQ treatment regardless of treatment duration <180 days or ≥180 days (adjusted ORs 1.61, and 1.44; p = 0.133 and 0.122, respectively), <50% or ≥50% adherence rate (adjusted ORs 1.62 and 1.37; p = 0.084 and 0.235, respectively) or daily dose of <400 mg or ≥400 mg (adjusted ORs 1.47 and 1.53; p = 0.096 and 0.210, respectively). In Table 3, we analyzed the sub-group for patients with ischaemic heart disease which found that the arrhythmia was not increased in HCQ treatment compared with non HCQ group (adjusted OR 1.1, 95% CI 0.298–4.08, p = 0.883). The sensitivity analysis about possible life-threatening ventricular arrhythmia is shown in Fig 3. Similarly, the results showed that the risk of events was not associated with HCQ treatment (adjusted OR 1.02, 95% CI 0.19–5.41, p = 0.985).

**Table 3:** Subgroup multivariable logistic regression analysis of risk of arrhythmia in patients with ischaemic heart disease.

	HCQ		Non-HCQ		Adjusted OR		
	N	No. of arrhythmia	N	No. of arrhythmia		95% C.I.	p-value
Ischaemic heart disease							
No^†^	172	90	257	107	1.49	0.997–2.21	0.052
Yes^‡^	19	14	54	40	1.10	0.298–4.08	0.883

HCQ: hydroxychloroquine. COPD: chronic obstructive pulmonary disease.

†Adjusted for HCQ, hypertension, hyperlipidemia, chronic liver disease, chronic kidney disease, diabetes, COPD, stroke, and heart failure.

‡Adjusted for HCQ, hypertension, hyperlipidemia, chronic liver disease, diabetes, COPD, and stroke.

## Discussion

The main purpose of this study was to clarify the controversy concerning the safety of HCQ treatment and the risk of cardiac arrhythmia in patients with SLE. The results indicated that the SLE patients using HCQ did not have higher risks of all kinds of cardiac arrhythmias or conduction disturbance. Moreover, the risk of arrhythmia was not affected by a longer duration of HCQ treatment, higher daily dose or better drug adherence rate. To the best of our knowledge, this is the first study to use nationwide population-based data to assess the risk of arrhythmia and HCQ treatment in patients with SLE. In our recent cohort study, patients with RA using HCQ did not have a higher risk of cardiac arrhythmia regardless of the daily HCQ dose or follow-up duration [19], which is consistent with the current study.

Subsequently, observational and prospective studies have reported on the efficacy and safety of HCQ treatment for Covid-19. In an observational study with 1446 Covid-19 patients, Geleris et al. reported that HCQ treatment did not reduce the risk of intubation or death, but the reasons for mortality were not illustrated [20]. In their study, the HCQ group received significantly more of the macrolide antibiotic azithromycin even after propensity score matching, and multiple confounding factors such as underlying ischaemic heart disease, heart failure and cardiac arrhythmia were not adjusted. Baseline QTc interval and heart rate were also important concerns before treatment, and cardiac death caused by new-onset arrhythmia was one of the possible reasons for negating the benefit of HCQ and azithromycin treatment. In addition, Cavalcanti et al. conducted a multicentre, open-label, randomised controlled trial with 667 patients [21], and reported that the use of HCQ in patients with mild-to-moderate Covid-19 did not improve their clinical status at 15 days. They found that prolongation of QTc interval was more frequent in the HCQ group, but that there was no increase in arrhythmia. The relatively small sample size and low event rate may have affected the outcomes of their study. In the present study, we found no correlation between cardiac arrhythmia and the use of HCQ in SLE patients. The strength of this study lies in its large dataset of patients enrolled in the NHI system, which covers 99% of the Taiwanese population and reduces bias from selection, participation, or poor recall [22]. Prospective clinical trials with a long follow-up period are difficult to perform quickly to answer our study hypothesis, which is important for the clinical application of HCQ.

Although the risk of TdP with baseline QTc or drug-induced prolongation of the QTc interval is not a linear relationship, it is a potentially lethal polymorphic ventricular tachycardia that can cause sudden cardiac death. Previous case reports have described associations between HCQ and QTc prolongation and ventricular arrhythmia [12, 14, 15]. However, other studies have reported no significant differences in QTc interval and heart rate between normal values and patients treated with HCQ [23]. The main cardiac arrhythmia in our study was defined as a new diagnosis of all arrhythmias including conduction disorder, sinus node dysfunction, supraventricular and ventricular tachyarrhythmias. The greatest safety concern with HCQ is due to its action on iKr channels, causing QTc prolongation which may result in life-threatening ventricular arrhythmia and TdP. The sensitivity sub-analysis of only ventricular tachyarrhythmias also disclosed consistent outcomes. Our data showed that the administration of HCQ had a neutral effect on the development of all arrhythmias and especially ventricular tachyarrhythmias.

Regarding comorbidities, ischemic heart disease was associated with a significantly increased risk of arrhythmia, but the other comorbidities did not (Table 2). This anticipated result is likely due to acute or chronic ischaemic heart disease related to various arrhythmogenic alterations. A previous study reported that the cardiotoxicity of HCQ was possibly associated with the cumulative dose [24], and that conduction disorders including atrioventricular block, complete heart block, and bundle branch block are the main cardiac side effects [24]. The chronic use of HCQ has also been reported to provoke cardiac arrhythmia [15]. However our additional analysis disclosed HCQ did not increase arrhythmia whether in patients with ischemic heart disease or not (Table 3). Further sub-analysis did not show an increase in cardiac arrhythmia with a longer duration of HCQ use of ≥180 days, higher HCQ dose of ≥400 mg or better adherence (Fig 2). These results are also consistence with our previous report [19]. These data suggest the safety of HCQ regardless of the different cumulative dose and that there is no dose-dependent effect with long-term treatment for rheumatic diseases such as SLE and RA.

There are several limitations to this study. First, data on QTc interval as assessed by electrocardiography (ECG) were not available in the LHID, although ECG is recommended to estimate the QTc interval in individuals receiving HCQ [25]. Nevertheless, we believe that our results are robust with regards to the risk of arrhythmia with HCQ therapy. Second, we did not investigate other drugs that could have prolonged QT, and this may have affected the results. Third, we did not have access to the results of serum blood tests, such as potassium, calcium, magnesium, glucose, or thyroid function, which may influence the development of arrhythmia. Fourth, our disease definition was based on codified data without any procedure codes, which may have led to over diagnosis. Therefore, we added SLE with at least two outpatient clinic visits or one admission to reduce this bias. Fifth, our study population consisted of patients with SLE, hence our results may not be totally applicable to patients who use HCQ for other diseases. Sixth, some baseline comorbidities were not balanced in the two groups. The study sample size was not large enough for further propensity score matching, as few SLE patients had arrhythmia (only 258 events of new arrhythmia in patients with underlying SLE among a population of one million). Finally, this study was hypothesis driven, so some unknown bias could exist. A larger randomised control trial is necessary to verify the outcomes of this study.

## Conclusion

HCQ treatment in patients with SLE did not significantly increase all types of cardiac arrhythmias or ventricular tachyarrhythmias regardless of the higher cumulative dose. This neutral outcome may clarify the safety of HCQ regarding arrhythmias.
